# Entrance and Survival of *Brucella pinnipedialis* Hooded Seal Strain in Human Macrophages and Epithelial Cells

**DOI:** 10.1371/journal.pone.0084861

**Published:** 2013-12-20

**Authors:** Anett K. Larsen, Ingebjørg H. Nymo, Benjamin Briquemont, Karen K. Sørensen, Jacques Godfroid

**Affiliations:** 1 Section for Arctic Veterinary Medicine, Department of Food Safety and Infection Biology, Norwegian School of Veterinary Science, Tromsø, Norway; 2 The Fram Centre, High North Research Centre for Climate and the Environment, Tromsø, Norway; 3 Faculty of Science, Catholic University of Louvain, Louvain-la-Neuve, Belgium; 4 Vascular Biology Research Group, Department of Medical Biology, University of Tromsø, Tromsø, Norway; East Carolina University School of Medicine, United States of America

## Abstract

Marine mammal *Brucella* spp. have been isolated from pinnipeds (*B. pinnipedialis*) and cetaceans (*B. ceti*) from around the world. Although the zoonotic potential of marine mammal brucellae is largely unknown, reports of human disease exist. There are few studies of the mechanisms of bacterial intracellular invasion and multiplication involving the marine mammal *Brucella* spp. We examined the infective capacity of two genetically different *B. pinnipedialis* strains (reference strain; NTCT 12890 and a hooded seal isolate; B17) by measuring the ability of the bacteria to enter and replicate in cultured phagocytes and epithelial cells. Human macrophage-like cells (THP-1), two murine macrophage cell lines (RAW264.7 and J774A.1), and a human malignant epithelial cell line (HeLa S3) were challenged with bacteria in a gentamicin protection assay. Our results show that *B. pinnipedialis* is internalized, but is then gradually eliminated during the next 72 – 96 hours. Confocal microscopy revealed that intracellular *B. pinnipedialis* hooded seal strain colocalized with lysosomal compartments at 1.5 and 24 hours after infection. Intracellular presence of *B. pinnipedialis* hooded seal strain was verified by transmission electron microscopy. By using a cholesterol-scavenging lipid inhibitor, entrance of *B. pinnipedialis* hooded seal strain in human macrophages was significantly reduced by 65.8 % (± 17.3), suggesting involvement of lipid-rafts in intracellular entry. Murine macrophages invaded by *B. pinnipedialis* do not release nitric oxide (NO) and intracellular bacterial presence does not induce cell death. In summary, *B. pinnipedialis* hooded seal strain can enter human and murine macrophages, as well as human epithelial cells. Intracellular entry of *B. pinnipedialis* hooded seal strain involves, but seems not to be limited to, lipid-rafts in human macrophages. *Brucella pinnipedialis* does not multiply or survive for prolonged periods intracellulary.

## Introduction

Brucellosis is an infectious disease that affects a wide range of mammalian species and is regarded as the world’s most common bacterial zoonotic disease [1]. For decades, the genus *Brucella* included six species with different preferred terrestrial mammalian hosts, four of which are pathogenic for humans [2]. Recently four additional species have been included [3–5]. The occurrence of human disease is dependent on animal reservoirs, including wildlife [6].

*Brucella* spp. were isolated from marine mammals for the first time in 1994 [7,8] and validly published as members of genus *Brucella* with the names *B. pinnipedialis* (pinnipeds; seals, sea lions and walruses) and *B. ceti* (cetaceans; whales, dolphins, and porpoises) in 2007 [3]. Marine mammal brucellae have since been serologically indicated in and isolated from pinnipeds and cetaceans from multiple locations; however gross pathology in association with *Brucella* infection in marine mammals is exclusively found in cetaceans [9,10]. The results from experimental infections in various animal species are diverging and the zoonotic potential of the marine mammal brucellae is largely unknown [7,11–13]. However, reports of human disease exist [14–16]. Interestingly, none of the naturally infected human cases reported contact with marine mammals, but consumption of raw seafood was noted [15,16]. All three *Brucella*-isolates from the naturally acquired infections shared an identical genotype (sequence type 27), to date only being found in marine mammals in Pacific waters [17]. The infectious cycle of marine mammal brucellae is to a large extent uncertain and unknown hosts or carriers may exist. *Brucella melitensis* has been isolated from Nile catfish (*Clarias gariepinus*) [18], while *B*. *ceti* and *B. pinnipedialis* have been isolated from lungworms in cetaceans [19] and pinnipeds [20] respectively, pointing at other possible reservoirs in the marine ecosystem besides pinnipeds and cetaceans. In light of the extensive use of marine resources, including products from marine mammals, increased knowledge about *Brucella* spp. in the marine environment and their possible implications in human disease, is an important issue in the “One Health” perspective. 

*Brucella* spp. are facultative intracellular bacteria that can survive and replicate within membrane-bound compartments in phagocytes and epithelial cells [21–23]. Studies of the mechanisms of bacterial invasion and intracellular multiplication involving the marine mammal brucellae are sparse and previously only investigated by Maquart and co-workers (2009). They observed different infection dynamics between the marine mammal brucellae during macrophage infection *in vitro* [24]. *Brucella pinnipedialis* reference strain (NCTC 12890), isolated from a common seal (*Phoca vitulina*), was able to infect and multiply in human and murine macrophage cell lines to the same extent as the virulent terrestrial *Brucella* spp. In contrast, *B. pinnipedialis* isolated from hooded seal (*Cystophora cristata*) showed absence of invasive potential. This particular strain displayed a high prevalence in the declined Northeast Atlantic hooded seal stock [25] and has been isolated from various organs of young seals; however *Brucella*-associated pathology has not been identified [26]. The Northeast Atlantic stock of hooded seal is now 10 – 15 % of the 1946 population and has been stable at this low level since the 1980s [27]. How *B. pinnipedialis* hooded seal strain (HS) may affect the population dynamics is unknown, as we lack knowledge about the strains ability to establish infection and cause pathology. Entering macrophages after a transient initial bacteremia protects brucellae from antibodies and complement during dissemination in the host [28]. It seemed intriguing that the hooded seal strain could be isolated from multiple organs [26] without being able to escape an immune response by entering host cells. Recent studies have now confirmed that *B. pinnipedialis* HS is able to enter hooded seal alveolar macrophages, but is eliminated within 48 h post infection [29]. Furthermore, an age-dependent pattern of anti-*Brucella* antibodies, indicating exposure early in life with a subsequent clearance of infection, is identified in both hooded [30] and harbor seals [31]. 

To increase our general knowledge about the marine members of a bacterial genus that occurs at the animal – human interface and to predict possible implications in human disease, extended information about intracellular entry, trafficking and multiplication of *B. pinnipedialis* in human cells should be obtained. The hooded seal strain is in whole genome analysis found to differ from the other marine brucellae with respect to genome size and GC content [32]. Knowing that the strains isolated from the human incidents also differ from the other marine *Brucella* spp., we aimed to study the infection biology of the hooded seal strain. The relationship between *B. pinnipedialis* HS and human macrophages was explored by adding an inhibitor study, as well as immunocytochemistry and transmission electron microscopy, to the conventional macrophage infection assays. Macrophage activation and cellular integrity following bacterial entry and elimination were investigated to further study the interaction of *B. pinnipedialis* with phagocytes and epithelial cells. 

## Materials and Methods

### Reagents and media

Gentamicin, penicillin-streptomycin, phorbol 12-myristate 13-acetate (PMA), β-methyl cyclodextrin, Dulbecco’s minimum essential medium, RPMI 1640, Dulbecco’s Modified Eagle’s Medium/Nutrient Mixture F-12 Ham (DMEM/F12), Triton X-100 and lipopolysaccharide (LPS) from *Escherichia coli* (O111:B4) were all purchased from Sigma-Aldrich, St. Louis, USA. Fetal bovine serum (FBS) was from Gibco, Life Technologies, Paisley, UK. Tryptic soy agar (TSA) was from Merck Millipore, Darmstadt, Germany and sheep blood agar was from Oxoid, Oslo, Norway. Rabbit polyclonal anti-*Brucella* antibody was kindly provided by Prof. J.J. Letesson, Faculté Universitaire Notre Dame de la Paix, Namur, Belgium. Alexa Fluor 488 goat-anti-rabbit IgG and Lysotracker Red were from Molecular Probes, Life Technologies, Paisley, UK. Nuclear dye DRAQ5 was purchased from Cell Signaling, Danvers, USA. Fluorescence mounting medium was from Dako, Glostrup, Denmark. *Brucella abortus* antiserum was obtained from Remel Europe Ltd., Kent, UK. The CytoTox 96 Non-Radioactive Cytotoxicity Assay and the Griess Reagent System were from Promega, Madison, USA.

### Bacterial strains and growth conditions

The *Brucella* strains used were *B. suis* 1330 (ATCC no. 23444/NCTC 10316) (provided by Dr. B. Djønne, Norwegian Veterinary Institute, Oslo, Norway), *B. pinnipedialis* reference strain (NCTC 12890) (provided by Dr. G. Foster, Scottish Agricultural College, Consulting Veterinary Services, a part of Scotland’s Rural College, Inverness, UK) and a *B. pinnipedialis* hooded seal isolate (animal number 17, spleen, ref [26]; from now on entitled B17). The strains were kept at -80 °C on Microbank™ beads (Pro-Lab Diagnostics, Round Rock, USA). Before each assay a bead was taken from one specific Microbank™ batch and plated on sheep blood agar at 37 °C in a 5 % CO_2_ atmosphere for 2 – 4 days, the strains were thereafter plated again on sheep blood agar and grown at 37 °C in a 5 % CO_2_ atmosphere for 48 h for *B. suis* 1330 and 96 h for *B. pinnipedialis*. Dilutions of the bacteria in sterile PBS were used to prepare the final infective solution. We verified the expression of smooth surface antigens for the Microbank™ batches by crystal violet staining and agglutination with antiserum to smooth *B. abortus* [33,34]. The identity of the strains was verified before and after the gentamicin protection assay by PCR and gel electrophoresis with the marine mammal brucellae specific primers designed to amplify the *bp26* gene [35] and the primer sets Bruce 11, 18 and 45 from the MLVA-15 assay [36,37].

### Cell cultures

THP-1 (ATCC no. TIB-202), a human monocyte/macrophage-like cell line, was cultured in RPMI 1640 and HeLa S3 cells (ATCC no. CCL-2.2) were kept in DMEM/F12. The murine macrophage cell lines, RAW264.7 (ATCC no. TIB-71) and J774A.1 (ATCC no. TIB-67), were cultured in DMEM. To avoid prolonged exposure to trypsin treatment, a cell scraper was used to detach the adherent macrophage cell lines when passaged. All culture media were supplemented with 10 % fetal bovine serum, 100 IU/ml penicillin, and 100 μg/ml streptomycin. 

### Gentamicin protection assay

THP-1 cells were differentiated into macrophage-like cells by the use of PMA as previously described [38]. In brief, THP-1 cells were seeded (3.5 x 10^5^ cells/well) in 24 well plates and cultured for 24 h in complete RPMI containing 50 ng/ml PMA. RAW264.7, J774A.1 and HeLa S3 cells were seeded (2 x 10^5^ cells/well) in 24 well plates and cultured in the appropriate complete medium for 24 h prior to infection. The cells were maintained in their respective culture medium without penicillin/streptomycin during the *Brucella* infection assays. The macrophage cell lines were challenged with *Brucella* spp. with a multiplicity of infection (MOI) of 50 and the HeLa cells with a MOI of 500 for 1 h at 37 °C, supplemented with 5 % CO_2_. The exposure was synchronized by centrifugation at 230 x g for 10 min in room temperature (RT). This stage was ended by first rinsing the wells twice with PBS, and refilling with 1 ml of complete culture medium containing 100 µg/ml gentamicin to kill extracellular bacteria. After 1 h the medium was replaced with complete culture medium containing 10 µg/ml gentamicin, and the cells were incubated for the desired period of time (1.5, 7, 24, 48, 72, and 96 h). Before harvesting of intracellular bacteria the cells were washed three times with PBS to remove remaining antibiotics. The cell membranes were disrupted using 300 µl/well of 0.1 % Triton X-100 in PBS followed by incubation at 37 °C for 10 min. Sterile cell scrapers were used to ensure complete detachment of all cells and the lysate was repeatedly pipetted to aid cell membrane disruption. TSA agar plates were inoculated in duplicate with 100 µl each of lysate in serial dilutions and evaluated for the presence of colony forming units (CFU)/well. Supernatants were also plated to control the efficiency of extracellular bacterial killing by gentamicin. The MOI applied was verified for each assay. 

### Immunocytochemistry

THP-1 and HeLa cells were seeded on glass coverslips in 12 well plates (2.0 – 2.5 x 10^5^ cells/well). The THP-1 cells were PMA-treated for differentiation. After 24 h the cells were challenged with B17 at a MOI of 50 (THP-1) or 500 (HeLa) as described in the gentamicin protection assay. Infected cells were incubated with 75 nM LysoTracker Red for 1 h prior to fixation. The cells were fixed for 15 min at RT using 4 % paraformaldehyde, 0.02 M sucrose, in PBS, pH 7.2 at desired time points following bacterial exposure and washed once in PBS before permabilization in 0.1 % Triton X-100 for 4 min. Immune labeling was done using rabbit polyclonal anti-*Brucella* spp. antibody, diluted at 1:100. Secondary antibody was Alexa Fluor 488 goat-anti-rabbit IgG, diluted at 1:500. The fluorescent DNA dye DRAQ5, diluted at 1:1000, was used for visualization of cell nuclei. Confocal microscopy was performed using a Zeiss LSM510 META system (Carl Zeiss, Obercochen, Germany) equipped with a 40X 1.2NA water immersion lens. Three sequential channels were recorded using the following excitation and emission parameters: Alexa 488 was excited at 488 nm and fluorescence collected through a 500 – 550 nm BP filter; LysoTracker Red was excited at 543 nm and fluorescence collected through a 565 – 615 nm BP filter; DRAQ5 was excited at 633 nm and fluorescence collected in the META detector from 644 – 700 nm.

### Immune transmission electron microscopy

THP-1 and HeLa S3 cells were seeded in 6 well plates at 3.5 x 10^5^ and 1.5 x 10^5^ cells/well, respectively, and allowed to grow for 48 h. The cells were challenged with B17 as described for the gentamicin protection assay and fixed at 1.5, 7 and 24 h post infection using 4 % formaldehyde, 0.1 % glutaraldehyde in 100 mM sodium phosphate buffer, pH 7.2. Samples were processed for cryoimmune electron microscopy as previously described [39]. Cryosections were labeled using rabbit polyclonal anti-*Brucella* antibody, diluted at 1:80. Positive labeling was detected by 10 nm protein A-gold complexes. The dried sections were examined in a JEOL JEM 1010 transmission electron microscope (JEOL, Tokyo, Japan) operating at 80 kV. Control experiments were routinely included in parallel by omission of the primary antibodies. Sections of fixed B17 functioned as positive control.

### Inhibitor assays

Monolayers of PMA-treated THP-1 cells (3.5 x 10^5^ cells/well) were cultured in 24 well plates and pre-incubated for 45 min at 37 °C in complete culture medium containing increasing concentrations of the cholesterol-scavenging drug β-methyl cyclodextrin, as previously described [40]. After inhibitor treatment, macrophages were washed once with complete culture medium and infected with *Brucella* spp. (MOI = 50). Bacterial uptake was determined using the gentamicin protection assay. 

### Cellular integrity and macrophage activation

Potential toxic cell damage due to inhibitor treatment or *Brucella* spp. infection was measured by quantitatively determining the release of lactate dehydrogenase using the CytoTox 96 Non-Radioactive Cytotoxicity Assay. Production of nitric oxide (NO) following infection, indicating macrophage activation, was assessed using the Griess Reagent System. Both assays were performed according to the manufacturer’s instructions. Absorbances were read using an Epoch Microplate Spectrophotometer (BioTek, Winooski, USA). LPS from *Escherichia coli* (O111:B4) at 0.5, 1, and 10 µg/ml was used as a positive control for NO measurements.

### Statistical analysis

Group data were compared using Student’s *t* test for independent samples. Differences were considered significant for *p* values of < 0.05.

## Results

### Verification of the identity of the strains

PCR and gel electrophoresis showed bands of the expected size for all strains used in the study [35–37] (data not shown). All strains expressed smooth surface antigens before and after execution of the gentamicin protection assay.

### *Brucella pinnipedialis* HS enters macrophages and epithelial cells in vitro

**Figure 1:**
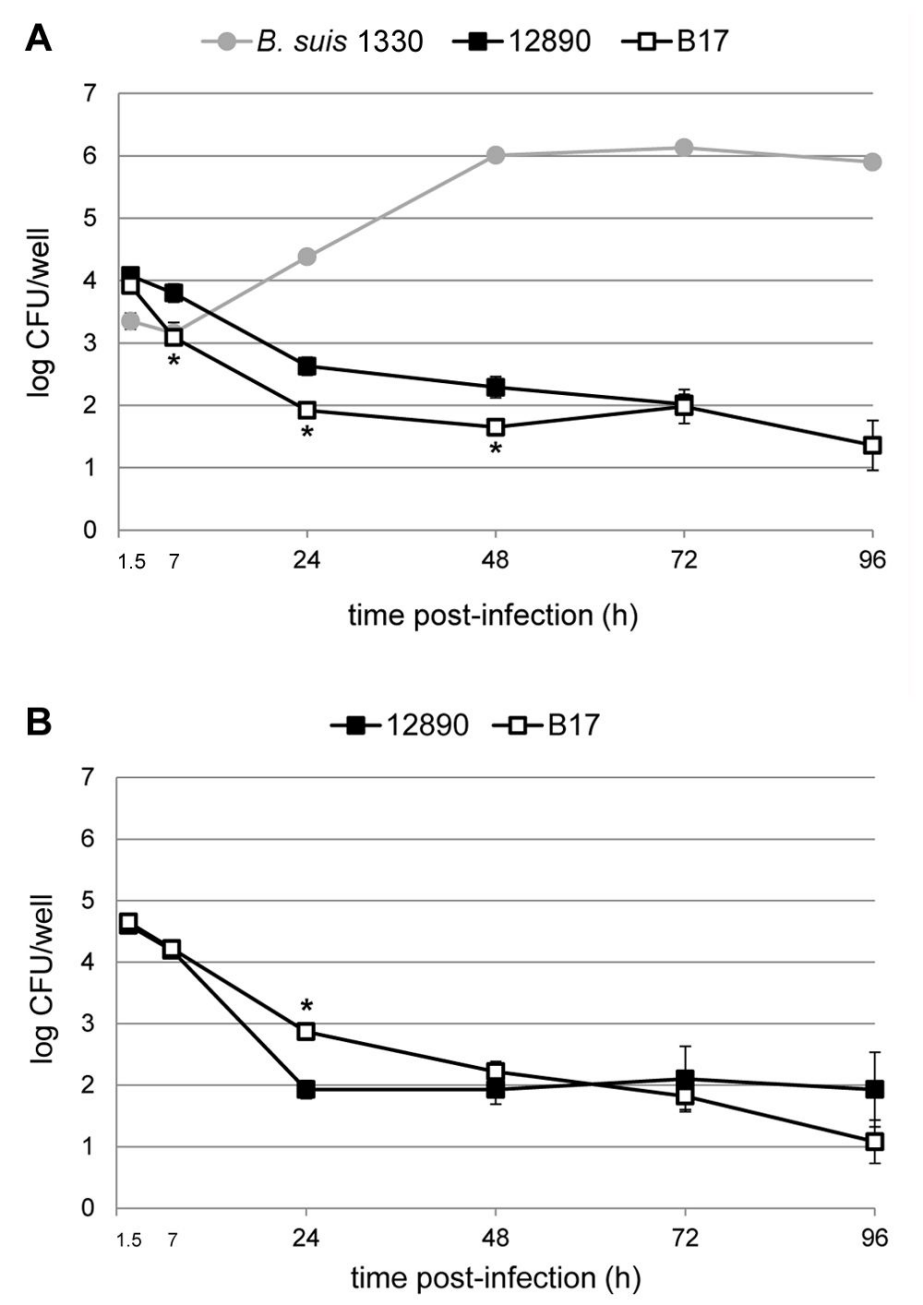
Infection dynamics of *Brucella* spp. in human macrophages and HeLa cells. THP-1 cells (A) and HeLa S3 cells (B) were challenged with *B. pinnipedialis* HS (B17), *B. pinnipedialis* reference strain (12890; harbor seal) and *B. suis* 1330 (only THP-1 cells) in a gentamicin protection assay. Both *B. pinnipedialis* strains tested entered in similar numbers in the respective cell types. None of the marine mammal *Brucella* strains were able to multiply within 48 h pi, as observed for *B. suis* 1330. At 72 and 96 h pi, slightly higher numbers of bacteria could be retrieved from certain wells, most pronounced with the reference strain (12890) in HeLa cells, but none reached the initial numbers at 1.5 h pi. Error bars correspond to the standard error. Each indicator represents the mean of four (*B. suis* 1330), six (12890) or nine (B17) replicate wells from 2 or 3 separate assays in A, and six replicate wells from 2 separate assays (B17 and 12890) in B. * (different from 12890, *p* < 0.05).

**Figure 2:**
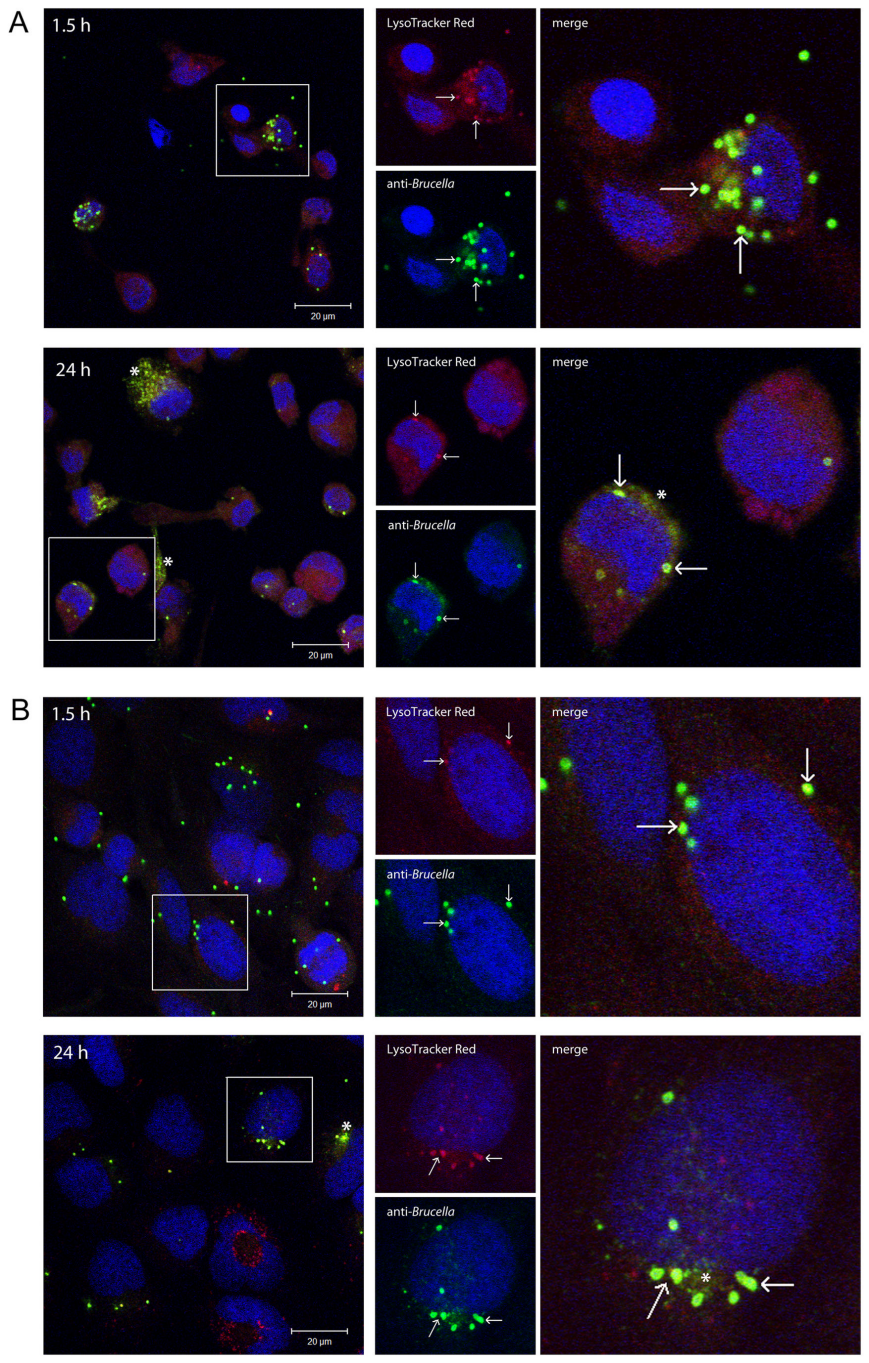
Intracellular localization of *B*. *pinnipedialis* HS. THP-1 (panel A) and HeLa cells (panel B) were cultured on glass coverslips and challenged with *B. pinnipedialis* HS (B17) as described in the gentamicin protection assay. Cells were incubated with LysoTracker Red (red) for 1 h before fixed at 1.5 and 24 h after exposure and immune labeled with anti-*Brucella* antibody 1:100, and Alexa 488 goat-anti-rabbit 1:500 (green). DRAQ5 was used for visualization of the cell nuclei (blue). Confocal microscopy revealed colocalization (arrows) of B17 and lysosomal compartments in both cell lines at 1.5 h (weakly in the HeLa cells) and 24 h. Bacterial debris scattered throughout the cytoplasm was observed at 24 h pi (asterisks). Scale bars: 20 µm.

**Figure 3:**
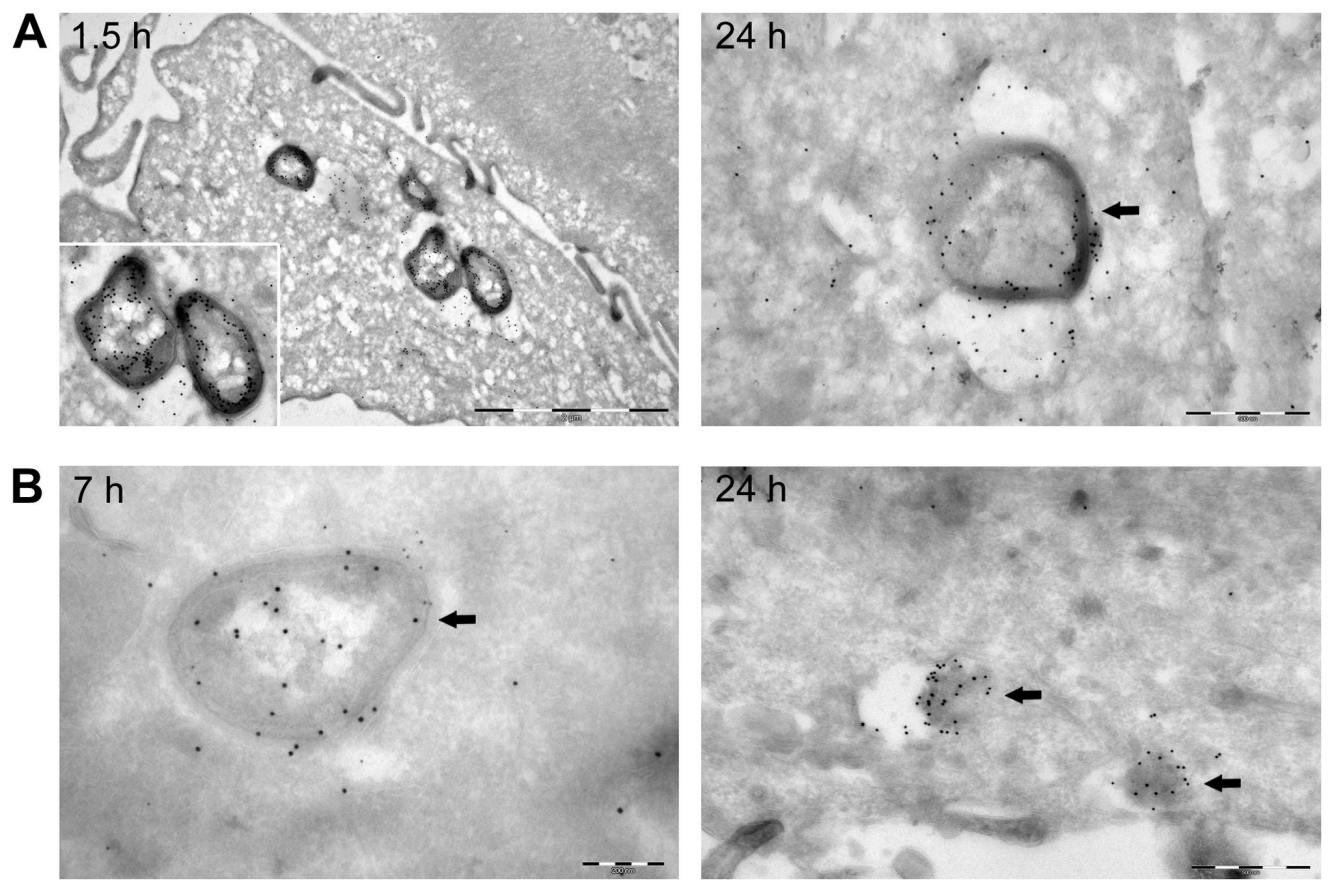
Cryoimmune electron microscopy of *B. pinnipedialis* HS in THP-1 and HeLa cells. Transmission electron micrographs showing uptake of *B. pinnipedialis* HS (B17) in THP-1 (A) and HeLa (B) cells. The cells were challenged for 1 h with bacteria as described in the materials and method section and fixed at indicated time points post infection. Bacteria or bacterial debris were labeled with a rabbit anti-*Brucella* antibody, and 10 nm protein-A conjugated gold particles (arrows). A) Shows intact bacteria in THP-1 cells after chase periods of 1.5 h and 24 h, respectively. B) Shows intact bacteria in a HeLa cell after a 7 h chase. At 24 h post infection the bacterial structure was to a great extent destroyed and debris labeled with anti-*Brucella* antibody was detected in vacuolar structures (arrows in B, 24 h chase). Scale bars: A, 1.5 h: 2 µm; A, 24 h: 500 nm; B, 7 h: 200 nm; B, 24 h: 500 nm.

The results from the gentamicin protection assay revealed that both *B. pinnipedialis* strains (12890 and B17) were able to enter human macrophage-like cells in culture (THP-1) (Figure 1A). Challenging the cells with a MOI of 50 lead to the retrieval of 3.92 log CFU for B17 after 1.5 h, compared to 4.08 log CFU retrieval for the reference strain 12890. B17 were also taken up in two murine macrophage cell lines (RAW264.7 and J774A.1) with the retrieval of 4.28 log CFU and 3.50 log CFU at the 1.5 h time interval respectively (Figures S1 and S2). A known pathogenic strain; *B. suis* 1330, was included as a control for the ability of the macrophage cell types to support internalization as well as intracellular multiplication. While being challenged with the same MOI of 50, the amount of protected bacteria retrieved for *B. suis* 1330 after 1.5 h pi was 3.35 log CFU and 3.24 log CFU for THP-1 cells (Figure 1A) and RAW264.7 (Figure S1) respectively. Additionally, both 12890 and B17 entered HeLa cells. Challenging the cells with a MOI of 500 lead to a retrieval of 4.60 and 4.65 log CFU at 1.5 h pi respectively (Figure 1B). No bacterial growth was observed from the plated supernatant controls. The intracellular localization of B17 in THP-1 and HeLa cells was confirmed using confocal microscopy (Figure 2A and B, video S1). Immune labeling with a rabbit anti-*Brucella* antibody and Lysotracker Red revealed colocalization of intracellular bacteria with lysosomes at both 1.5 and 24 h pi. *Brucella* bacteria were observed in 37 % of the THP-1 cells at 1.5 h pi; 67 % of the infected cells had one bacteria, 15 % two bacteria, while 18 % had more than two bacteria (total THP-1 cells, *n* = 498). The corresponding numbers in HeLa cells were 61 % infected cells; 40 % had one bacteria, 21 % two bacteria, while 39 % had more than two bacteria (total HeLa cells, *n* = 151). Immune electron microscopy further verified intracellular presence of B17 in THP-1 and HeLa cells (Figure 3). 

### *Brucella pinnipedialis* HS is gradually eliminated from cultured macrophages and epithelial cells

Following cellular entry during infection in THP-1 cells, B17 showed a large drop in intracellular bacterial numbers with one logarithmic decrease at 7 h (3.09 log CFU) and additionally one logarithmic reduction at 24 h (1.92 log CFU) pi (Figure 1A). At 72 and 96 h pi bacteria were still present intracellulary in some wells, but were completely eliminated in others, dividing the results in two groups. At 72 h pi, three of total nine wells displayed elevated bacterial numbers with mean 2.98 log CFU in the elevated group versus mean 1.48 log CFU in the low group. At 96 h pi, three of total nine well were elevated with mean 3.12 log CFU versus mean 0.57 log CFU. Although the total mean log CFU decreased from 24 to 96 h pi, this effect caused a high standard deviation for the 72 and 96 h time points. In murine macrophages, intracellular B17 was completely eliminated by 48 h (J774A.1) and 72 h (RAW264.7) pi (Figures S2 and S1). The variability between wells at later stages of the infection was not observed in these latter cell lines. Contrary to published data [24], strain 12890 did not multiply intracellulary in THP-1 cells and displayed the same pattern as B17 but with less variability between wells at 72 h pi (Figure 1A). 

In HeLa cells, the intracellular bacterial numbers retrieved at 24 h pi were lowered with almost two logarithmic decreases for B17 (2.87 log CFU) and more than two logarithmic decreases for 12890 (1.93 log CFU) compared to the initial numbers at 1.5 h pi (Figure 1B). From 48 h pi the total mean log CFU retrieved slowly decreased for B17, however some wells had higher retrievable bacterial counts at 72 h (three of total nine; mean 2.65 log CFU versus mean 1.4 log CFU) and 96 h (one of total nine; 3.28 log CFU versus mean 0.8 log CFU) pi compared to others. For 12890, the total mean intracellular bacterial counts were similar from 24 h to 96 h pi, but due to inter well differences the standard deviations are large. At 72 h pi, six of total nine wells still displayed elevated bacterial numbers with mean 3.12 log CFU in the elevated group versus mean 0.06 log CFU in the low group. At 96 h pi, still five of total nine wells were elevated with mean 3.41 log CFU versus mean 0.09 log CFU.

Although B17 was statistically different from 12890 at various time points during infection (7, 24, and 48 h pi in the THP-1 cell line and 24 h pi in HeLa cells), the outcome of infection was similar. *Brucella suis* 1330, serving as a positive control, showed the classical infection pattern with a slight drop in CFU at 7 h, followed by high intracellular multiplication from 24 h reaching 6.1 log CFU after 72 h in the THP-1 cell line (Figure 1A). Intracellular multiplication of *B. suis* 1330 was also confirmed in murine macrophages (RAW264.7) (Figure S1).

### Intracellular *B. pinnipedialis* do not cause macrophage NO-production or increased cell death

Nitrite, as an indicator of NO-production, was not detected to be released from murine macrophages (RAW264.7) challenged with *B. pinnipedialis* reference strain (12890) or HS (B17) at 1.5, 24 or 48 h pi. Measurements of nitrite were not found applicable in the THP-1 cell line, this in line with previous reports [41]. No release of lactate dehydrogenase was detected following infection with either 12890 or B17, suggesting minimal cytotoxicity induced by intracellular bacterial localization and degradation (results not shown). 

### Internalization of *B. pinnipedialis* HS depends on lipid raft-associated mechanisms

**Figure 4:**
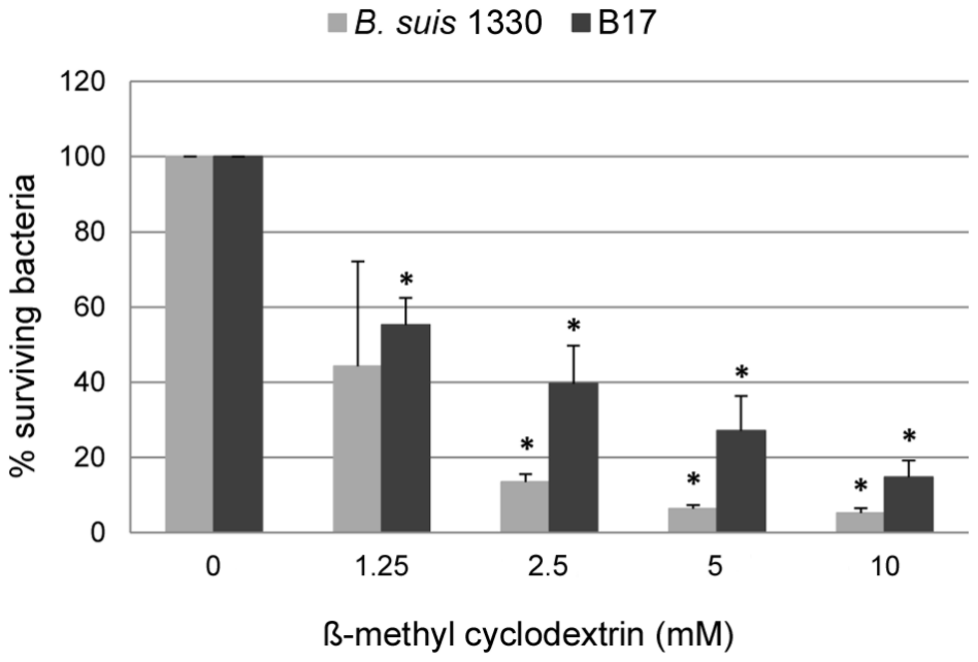
Effect of β-methyl cyclodextrin on human macrophage infection with *B. suis* 1330 and *B. pinnipedialis* HS. THP-1 cells (3.5 x 10^5^/well) were seeded in 24 well plates and treated with 50 ng/ml PMA for 24 h. Adherent cells were pre-incubated for 45 min in complete medium containing increasing concentrations of β-methyl cyclodextrin. After treatment, the cells were challenged with *B. suis* 1330 or *B. pinnipedialis* HS (B17) at a MOI of 50 and the number of viable intracellular bacteria at 1.5 h after exposure was determined using the gentamicin protection assay. The results are depicted as present surviving bacteria in inhibitor treated cells compared to infected non-inhibitor treated cells. Error bars correspond to the standard error. Each indicator represents the mean of three replicate wells from one assay (*B. suis* 1330) or six (B17) replicate wells from two separate assays. * (different from infected non-inhibitor treated cells, *p* < 0.05).

Marine mammal brucellae express a smooth type lipopolysaccharide (LPS) [42,43]. To evaluate if entry of B17 into human macrophages was associated with lipid raft components as reported for virulent terrestrial smooth *Brucella* spp. [44–47], THP-1 cells were pretreated with a cholesterol-scavenging drug (β-methyl cyclodextrin) before carrying out the gentamicin protection assay. *Brucella suis* 1330 was incorporated as a positive control showing more than 90 % reduction in retrieved CFU in the two highest concentrations of inhibitor (10 and 5 mM), and a 87 % reduction in the 2.5 mM concentration compared to untreated exposed cells measured after 1.5 h (Figure 4). Entrance of B17 was reduced with 85, 73, and 60 % respectively (Figure 4). No release of lactate dehydrogenase was detected following inhibitor treatment of the cells, suggesting minimal cytotoxicity induced by this procedure.

## Discussion

This work reports on the ability of *B. pinnipedialis* HS (B17) to enter human and murine macrophages, as well as an epithelial cell line *in vitro*. As these findings contrast the previous report regarding the behavior of hooded seal strains in human macrophages [24], our results from the gentamicin protection assays were verified by confocal and transmission immune electron microscopy. In line with the preceding report [24], *B. pinnipedialis* reference strain (12890) also entered human macrophages and HeLa cells, however, divergent to the prior report [24], the strain did not multiply intracellulary. When further exploring the interaction of *B. pinnipedialis* with phagocytes and epithelial cells, we revealed that although this marine mammal brucellae behave dissimilarly to pathogen terrestrial brucellae (lack of intracellular multiplication), it cannot be compared to engineered rough mutants with respect to macrophage entry and activation, or induction of cell death. 

Virulent smooth *Brucella* spp. as well as the naturally rough species internalize into macrophages via cholesterol-rich lipid rafts, including the raft-associated class A scavenger receptor (SR-A) [40,44–46]. This stealthy entry allows pathogenic *Brucella* spp. to limit early fusion with the endosome-lysosome pathway. Although internalized in low numbers, this enables the bacteria to activate their specific virulence genes involved in building a definite replication niche [48]. In order to gain more information regarding the possible pathogenicity of B17, a property that seems to be influenced by the mode of entry along with other bacterial traits, we explored if this marine brucellae has the capacity of stealthy entry. To this end we investigated the mode of B17 cellular entry mechanism by blocking lipid rafts. The number of bacteria entering inhibitor-treated cells was reduced with 85 % at the most. This was significantly different from untreated cells, but not as pronounced as for *B. suis* 1330 (more than 90 % reduction of bacterial cell entry). These results suggest that B17 can enter human macrophage-like cells via lipid raft- as well as non-lipid raft-associated mechanisms. Entering via lipid raft-associated mechanisms would give B17 an opportunity to activate possible virulence genes; however, the lack of such genes and/or cellular activation caused by other entry mechanisms may disrupt reaching or initiation of the replicative niche [49]. 

Although probably not the only determinant, bacterial mechanisms of cellular entry have been linked to the particular composition of LPS as different internalization mechanisms are observed for virulent smooth *Brucella* spp. and engineered rough mutants [48,50]. Recently, LPS from the smooth *B. suis* biovar 2 has been shown to lack some O-antigen epitopes previously thought to be present in all smooth brucellae [51,52]. The marine mammal *Brucella* spp. express a smooth-type LPS that show heterogeneity with regard to their O-polysaccharide common epitope content and average size [42,43]. B17 was previously shown to differ from the other marine mammal brucellae in whole genome analysis [32]. If structural differences in smooth strain LPS affect entry mechanisms, this could be a possible explanation for the variances observed between *B. suis* 1330 and B17 in the inhibitor assay. 

Compared to the pathogenic strains known to be the cause of human brucellosis (*B. melitensis/B. abortus/B. suis*), both B17 and 12890 entered the cell lines in higher numbers (range log CFU 3.92 – 4.65) than observed for *B. suis* (log CFU 3.35) in THP-1 cells. The number of intracellular bacteria is influenced by their mode of entry. In murine macrophages, *B. abortus* engineered rough mutants are showing a higher rate of infection compared to wild type *B. abortus*, both regarding the number of cells infected as well as the number of bacteria detected/cell [50]. This may be due to a more unspecific entry mechanism, as rough mutants are shown to enter via non-lipid raft associated mechanisms [48,50]. These rough mutants induce a strong degree of macrophage activation following infection, such as activation of NF-κB, increased expression and production of cytokines and inducible nitric oxide synthase (iNOS); an event that may lead to elimination of intracellular bacteria [47,49,53]. Additionally, multiplication of engineered rough mutants is attenuated due to their cytopathic effect on the macrophages, leading to cellular lysis and release of the bacteria, and not only due to intracellular killing as previously interpreted [50,54]. Confocal microscopy of THP-1 cells infected with B17 revealed a moderate rate of infected cells (37 %) at 1.5 h pi. Although certain individual cells were heavily loaded (> 10 bacteria/cell), only 18 % of the infected cells harbored more than 2 bacteria. This pattern resembles what is previously shown for wild type *B. abortus* [50], with a slightly higher rate of total infected cells. Along with the results from the inhibitor study, this supports the hypothesis of B17 entering the cells in similar manners as pathogenic terrestrial brucellae. The observed reduction of intracellular bacteria in the later stages after exposure for *B. pinnipedialis* HS is believed to be due to cellular activation and subsequent bacterial elimination, and not cell death. Morphological changes in the cell culture following exposure were not observed microscopically and release of lactate dehydrogenase was not detected following infection of the cells, suggesting minimal cytotoxicity induced by intracellular entry and degradation of bacteria. On the other hand, production of NO as a consequence of cell activation following infection with *B. pinnipedialis* HS in murine macrophages could not be detected. This in line with previous reports of *B. suis* shown not to induce expression of iNOS nor release of NO from murine macrophages [49]. The cellular response triggered by *B. pinnipedialis* is still unidentified, and future investigations including a broader panel of cytokines are suggested.

*In vitro*, virulent *Brucella* spp. ensure their survival by overriding the intracellular response following bacterial infection. By forming the *Brucella*-containing vacuole they are able to circumvent normal lysosomal destruction and facilitate intracellular proliferation [55–57]. This is demonstrated in *B. suis* infected THP-1 cells where the initial log value at 1.5 h (3.35 log CFU) was doubled by 48 h of infection (6.01 log CFU). Both B17 and 12890 were reduced quite rapidly the first 24 h after exposure in both macrophages and epithelial cells. Colocalization of B17 with lysosomal compartments was observed at both 1.5 and 24 h pi in THP-1 cells, weakly at 1.5 h and more pronounced at 24 h pi in HeLa cells. Additionally, at 24 h pi bacterial debris appears scattered throughout the infected cells indicating degradation of bacteria, as previously reported for an attenuated *B. abortus* strain [22,58]. Both findings support the observed reduction of intracellular bacteria retrieved at the later stages after infection. 

Although the infection dynamics of both B17 and 12890 displays a declining mean CFU, in some wells a higher number of intracellular bacteria were retrieved at 72 and 96 h than the corresponding numbers at 24 and 48 h pi. This shows that not all bacteria were eliminated equally efficiently, suggesting that in heavily bacterial-loaded cells a fraction of the intracellular bacteria may escape the lysosomal degradation process in order to proliferate intracellulary, as pointed out by others [59]. The observed divergence between wells was only noted in the THP-1 and HeLa cell lines, and not in the murine macrophages. This could reflect the bactericidal efficiency of the cell type, as HeLa cells are not professional phagocytes and PMA-induced THP-1 cells show increased macrophage maturation 5 days post treatment [60]. In this work, the gentamicin protection assay was initiated 24 h after PMA-treatment. Furthermore, activation of macrophages by either phorbol ester or 1.25-dihydroxyvitamin D_3_ is different [60,61] and caution should prevail when comparing results.

A relationship between MOI and a possible bacterial overload was not observed, as MOI 300, which was tested in the THP-1 cell line (results not shown), did not increase inter well differences compared to MOI 50, which was used for THP-1 cells throughout this study. 

In conclusion, this work shows that *B. pinnipedialis* HS is able to enter macrophages and HeLa cells *in vitro*. The entry mechanism in macrophages involves, but seems not to be limited to, membrane lipid rafts. Although able to survive for up to 96 h intracellulary, none of the strains were able to multiply at levels comparable to *B. suis* 1330 in the cell lines tested. A lack of/or low capacity to replicate and survive for prolonged periods within host cells, particularly macrophages, abolishes the ability to produce chronic infections as reported for terrestrial pathogenic brucellae [28]. The observed elevated bacterial numbers in some wells, most pronounced in HeLa cells, could imply that *B. pinnipedialis* may cause acute, transient infections in some cell types. An increased rate of survival in cell lines with reduced bactericidal competence gives room to speculate if changes in the cellular immune competence might support different infection dynamics.

In the Arctic region seal meat and other raw nutriments from marine mammals are consumed and for indigenous people it constitutes a large part of the diet. Consumption of raw bone marrow from reindeer has been documented to be a source of *B. suis* biovar 4 contamination in Alaska [62]. Reports of brucellosis in this area are sparse [63], however there may be an underreporting due to the unspecific symptoms of human brucellosis and non-diagnosed incidents could possibly occur [64]. In light of the close relationship to the marine environment and exposure to marine species/products, either occupational, or by lifestyle, further understanding of the pathogenesis of marine mammal brucellae in target species, as well as humans, is warranted. Although the results presented here indicate a low zoonotic potential of the strains tested, variations between strains as well as host immunity may influence the outcome of infection. Future research should aim to identify potential virulent traits in marine mammal brucellae that may have implications in the establishment of disease in marine mammals and humans. 

## Supporting Information

Figure S1 **Infection dynamics of *Brucella* spp. in murine macrophages.** RAW264.7 cells were challenged with *B. suis* 1330 and *B. pinnipedialis* HS (B17) in a gentamicin protection assay as described in materials and methods. B17 entered murine macrophages but was not able to multiply. Intracellular bacteria were eliminated within 72 h pi. *Brucella suis* 1330 showed the classical infection pattern with intracellular entry, a slight drop in intracellular bacterial numbers at 7 h pi and multiplication from 24 h pi. Error bars correspond to the standard error. Each indicator represents the mean of four replicate wells from one assay (*B. suis* 1330) or six replicate wells from two separate assays (B17).
(TIF)

Figure S2 **Infection dynamics of *B. pinnipedialis* HS in murine macrophages.** J774A.1 cells were challenged with *B. pinnipedialis* HS (B17) in a gentamicin protection assay as described in materials and methods. B17 entered murine macrophages but was not able to multiply. Intracellular bacteria were eliminated within 48 h pi. Error bars correspond to the standard error. Each indicator represents the mean of three replicate wells from one representative assay.
(TIF)

Video S1 ***Brucella pinnipedialis* HS in human macrophages.** THP-1 cells were cultured on glass coverslips and challenged with *B. pinnipedialis* HS (B17) in a gentamicin protection assay as described in the materials and method section. Cells were incubated with LysoTracker Red (red) for 1 h before fixed at 24 h after exposure and immune labeled with anti-*Brucella* antibody 1:100, and Alexa 488 goat-anti-rabbit 1:500 (green). DRAQ5 was used for visualization of the cell nuclei (blue). Three-dimensional animation of a confocal z-stack spanning the central volume of an infected macrophage, showing colocalization between bacteria and lysosomes at 24 h pi. Bacterial cells are situated at different levels intracellulary, many near the THP-1 nucleus. The animation was created in Volocity ver. 6.2.1 (Perkin-Elmer).
(MOV)
