# Supplementary figures and images for: Entrance and Survival of Brucella pinnipedialis Hooded Seal Strain in Human Macrophages and Epithelial Cells
Source: PLoS One. 2013 Dec 20;8(12):e84861. doi: 10.1371/journal.pone.0084861 (PMC3869908; doi:10.1371/journal.pone.0084861)

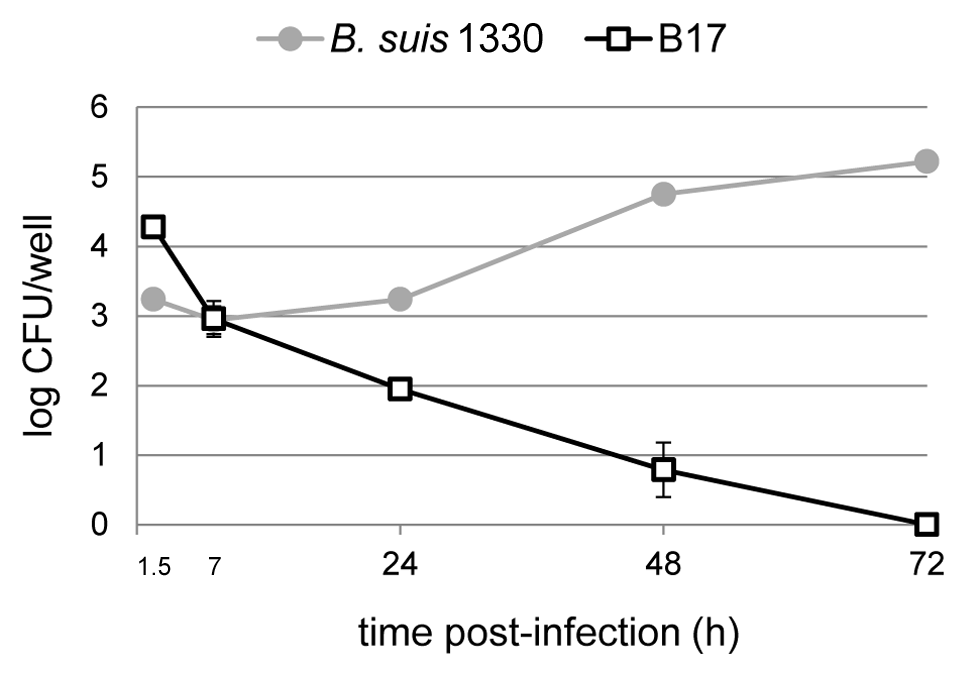

Supplement: Figure S1 — Infection dynamics of Brucella spp. in murine macrophages. RAW264.7 cells were challenged with B. suis 1330 and B. pinnipedialis HS (B17) in a gentamicin protection assay as described in materials and methods. B17 entered murine macrophages but was not able to multiply. Intracellular bacteria were eliminated within 72 h pi. Brucella suis 1330 showed the classical infection pattern with intracellular entry, a slight drop in intracellular bacterial numbers at 7 h pi and multiplication from 24 h pi. Error bars correspond to the standard error. Each indicator represents the mean of four replicate wells from one assay (B. suis 1330) or six replicate wells from two separate assays (B17). (TIF) [file pone.0084861.s001.tif]

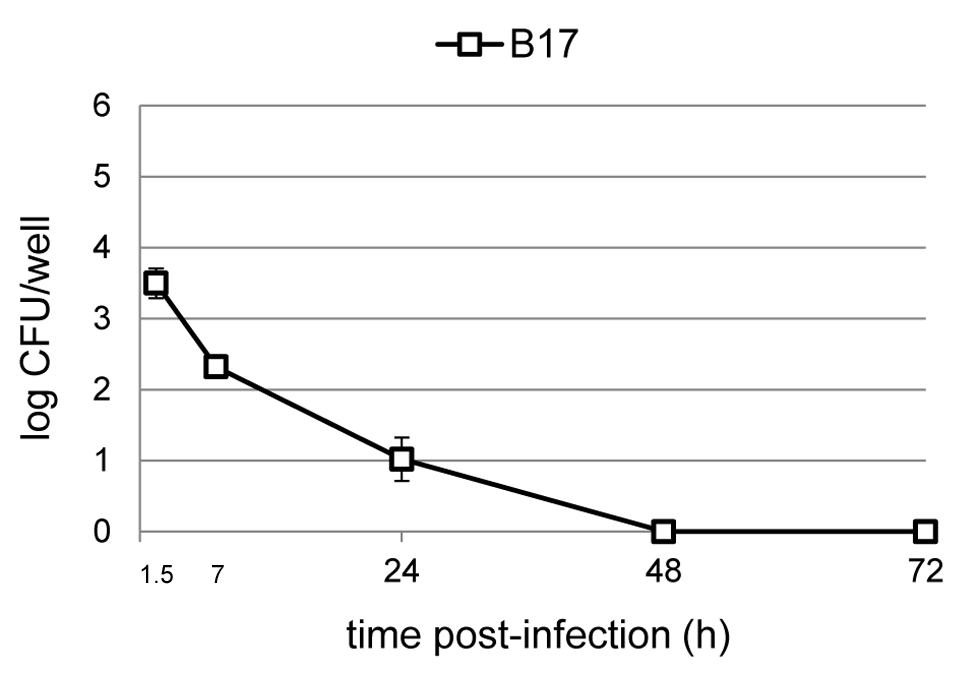

Supplement: Figure S2 — Infection dynamics of B. pinnipedialis HS in murine macrophages. J774A.1 cells were challenged with B. pinnipedialis HS (B17) in a gentamicin protection assay as described in materials and methods. B17 entered murine macrophages but was not able to multiply. Intracellular bacteria were eliminated within 48 h pi. Error bars correspond to the standard error. Each indicator represents the mean of three replicate wells from one representative assay. (TIF) [file pone.0084861.s002.tif]
